# Targeted immunotherapy for glioblastoma involving whole tumor-derived autologous cells in the upfront setting after craniotomy

**DOI:** 10.1007/s11060-023-04491-4

**Published:** 2023-11-29

**Authors:** Carrie E. Andrews, Jenny Zilberberg, Raul Perez-Olle, Mark A. Exley, David W. Andrews

**Affiliations:** 1 Department of Neurological Surgery, Thomas Jefferson University, Philadelphia, PA 19107 USA; 2 Imvax, Inc., Philadelphia, PA 19602 USA

**Keywords:** Glioblastoma, Brain tumor, Immunotherapy, Cancer vaccine

## Abstract

**Purpose:**

To date, immunotherapeutic approaches in glioblastoma (GBM) have had limited clinical efficacy as compared to other solid tumors. Here we explore autologous cell treatments that have the potential to circumvent treatment resistance to immunotherapy for GBM.

**Methods:**

We performed literature review and assessed clinical outcomes in phase 1 safety trials as well as phase 2 and 3 autologously-derived vaccines for the treatment of newly-diagnosed GBM. In one recent review of over 3,000 neuro-oncology phase 2 and phase 3 clinical trials, most trials were nonblinded (92%), single group (65%), nonrandomized (51%) and almost half were GBM trials. Only 10% involved a biologic and only 2.2% involved a double-blind randomized trial design.

**Results:**

With this comparative literature review we conclude that our autologous cell product is uniquely antigen-inclusive and antigen-agnostic with a promising safety profile as well as unexpected clinical efficacy in our published phase 1b trial. We have since designed a rigorous double-blinded add-on placebo-controlled trial involving our implantable biologic drug device. We conclude that IGV-001 provides a novel immunotherapy platform for historically intransigent ndGBM in this ongoing phase 2b trial (NCT04485949).

## Immunotherapy and therapeutic cancer vaccines

Despite major treatment successes in specific types and stages of cancer, as well as steady incremental progress in treatment of an increasing number of cancers, a large proportion of patients have limited effective treatment options, and there is still abundant room for improvement of cancer therapeutics [1, 2]. Although there is a half-century of evidence supporting the principles of immunotherapy, only in the past decade has it had a clinical impact in terms of therapeutic benefit [3]. Advances range from monotherapies with chimeric antigen receptor (CAR)-T cells in specific hematological malignancies to broadly-acting checkpoint inhibition in metastatic melanoma and other previously untreatable cancers. Prior reviews have discussed the use of CAR-T cells, oncolytic viruses, or immune checkpoint inhibition for treatment of glioblastoma, although these therapies have limits in terms of generalizability and clinical implementation [4–6]. Often only a small subset of patients benefit from these treatments, which has limited their applicability and clinical adoption. In many cases it is unclear why only some patients exhibit a clinical response [3, 7], and the immunological understanding of the mechanisms by which clinically active immunotherapies work and which patients will benefit is only now beginning to emerge [8].

Along with greater understanding of the underlying immunology of the various immunotherapies has come increasing evidence of clinical benefit, including evidence of the benefit of cancer vaccines. Despite being one of the first immunotherapies to be attempted [9], therapeutic cancer vaccines including short and long peptides [10], DNA [11, 12], RNA [13], and autologous tumor-derived cells [14] are conspicuously absent from the therapeutic arsenal [3]. This is likely because, as opposed to successful prophylactic treatments [15], therapeutic cancer vaccines must induce an immune response to existing cancer cells that have survived prior therapies [16].

Unlike most other immunotherapies, cancer vaccines typically have minimal side effects, as they rely on the selection of highly immunogenic tumor antigens that are only expressed by cancer cells. Tumor antigens can be classified as tumor-associated antigens (TAAs) or tumor-specific antigens (TSAs) [10, 17]. TAAs are preferentially overexpressed on tumor cells but can be present in healthy cells, or they may be cancer/testis antigens that are only expressed by tumor cells and adult reproductive tissues. TSAs, conversely, are de novo epitopes expressed by oncoviruses and shared or individual-specific neoantigens encoded by somatic mutations [17]. Compared to cancer vaccines that use TAAs or TSAs to induce tumor-reactive T cells, whole tumor-derived approaches have the benefit of being truly antigen-inclusive. They most often utilize sizable amounts of resected tumor material, rather than small biopsies, avoiding exclusion of relevant antigens due to tumor heterogeneity or sampling error. They do not require prediction of linear peptide antigens and include post-translationally modified antigens, which can be important drivers of tumor growth but are not encoded in mutations and therefore are not covered by neoantigen-based approaches [14, 18–20]. Additionally, they can include innate immune stimuli, although these can be balanced by immunosuppressive tumor cell components if there is no limit to the release of tumor cell contents [21, 22]. Several clinical trials reporting evidence of efficacy for autologous tumor-based cancer vaccines have recently been conducted [14, 23]. We used PubMed to search for the terms “glioblastoma,” “newly diagnosed,” “vaccine,” and “immunotherapy” to search for relevant articles reporting phase 2 or 3 trials for review.

## Clinical response to autologous cancer vaccines in the treatment of glioblastoma

Although immunotherapy has been revolutionary in treatment of many solid tumors, it has had limited efficacy in the treatment of glioblastoma (GBM). Current standard of care (SOC) for GBM consists of maximal safe resection followed by radiotherapy (RT) and temozolomide (TMZ) [24, 25]. Despite therapy, prognosis is dismal, with median life expectancy of 14.6 months in the original Stupp trial [24]. The first randomized study of tumor treatment fields (2:1 TTF vs. RT/TMZ alone) improved median OS (mOS) to 20.9 months [26]. Although a clear improvement, TTF has not been widely adopted as SOC. Consequently, there is substantial room for improvement in treatment of GBM, and immunotherapy provides promise as a revolutionary therapeutic strategy [27]. To date, however, the results of various approaches including vaccination, oncolytic virus, and immune checkpoint inhibition trials have been disappointing [4]. There are multiple potential roadblocks to the efficacy of immunotherapy in GBM, including the immune-privileged nature of the central nervous system, an immunosuppressive milieu surrounding the tumor, the hypoxic and necrotic micro-environment, the immunosuppressive nature of radiation and TMZ, routine corticosteroid administration, and the heterogeneous nature of genetic mutations both between patients with GBM and within a given tumor [5, 28].

Despite these challenges, development of several therapeutic cancer vaccines has been attempted in GBM. Three main approaches have been clinically tested. Some vaccines for GBM are derived from resected tumor cells in the generation of autologous tumor cell vaccines. Secondly, monocytes are harvested via leukapheresis and differentiated ex vivo into monocyte-derived dendritic cells (DCs), which are loaded with antigens. Thirdly, tumor antigens are combined in adjuvant formulations [29].

The double-blinded randomized phase 2 trial of a monocyte-derived DC vaccine ICT-107, involving autologous DCs pulsed with six synthetic peptides targeting appropriate HLAs binding antigens given once weekly over 4 weeks, increased mOS of newly-diagnosed GBM (ndGBM) patients by 2 months compared to placebo control, although findings were not statistically significant [30]. Progression-free survival (PFS) in the intent to treat (ITT) population, however, was significantly increased in the ICT-107 cohort by 2.2 months. Another phase 2 trial of patients with ndGBM who had undergone fluorescence-guided resection with 5-aminolevulinic acid were treated simultaneously with RT/TMZ and tumor lysate-pulsed autologous DCs. T cell proliferation, IFN-g production, and number of IFN-g -producing cells were measured. There was no demonstrated benefit in terms of PFS, and PFS was not associated with assays of immune response [31]. AV-GBM-1 (Aivita Biomedical, Inc.), a vaccine formulated with autologous DCs pulsed with a lysate of irradiated autologous tumor-initiating cells and admixed with granulocyte‐macrophage colony‐stimulating factor as adjuvant, was also tested in a phase 2 trial in ndGBM patients. AV-GBM-1 was administered after the completion of concurrent RT/TMZ. Results of the trial showed good treatment tolerability, and although median PFS (10.4 months) was longer than historical benchmarks, no mOS improvement was noted (16.0 months) [32].

The only phase 3 study of a cancer vaccine in GBM to date has been the DCVax®-L trial, which combined SOC with an autologous tumor lysate-pulsed monocyte-derived DC vaccine administered concomitantly with TMZ after completion of surgery and RT. Three hundred thirty-one patients were randomized in a 2:1 fashion and data analysis was performed with an ITT model [33]. The trial’s design, methods, and report raise several issues, undermining the ability to derive meaningful conclusions. Notably, multiple changes occurred years after the trial ended, including use of external controls in the initial randomization of patients, modification of the primary endpoint (OS instead of PFS), addition of a new study population (patients with recurrent GBM), addition of unplanned analyses, and other post-hoc changes [33, 34].

Human cytomegalovirus (CMV) proteins have been shown to be expressed in over 90% of GBMs, providing a potential target for immunotherapy [35]. CMV pp65-loaded DCs have been developed to target the CMV antigen pp65. Four weeks after undergoing surgical resection and concurrent RT/TMZ, patients received vaccination with the CMV pp65-loaded DCs on day 23 of the 28-day TMZ cycle. Vaccines were then administered monthly for 6 to 12 months in conjunction with maintenance TMZ. Eleven enrolled patients received three or more vaccinations and therefore met the criteria for inclusion in analysis; compared to a historical control cohort of 23 patients, PFS in vaccinated patients was 25.3 versus 8.0 months [36].

Heat shock proteins (HSPs) are intracellular chaperones that deliver tumor proteins to cytotoxic T cells, causing cleavage and presentation of tumor antigens to activate immune responses [37]. Autologous HSPs have therefore been identified as a possible vaccination agent in GBM. In a phase 2 trial of Prophage^™^, an autologous HSP peptide complex-96 vaccine was administered after surgical resection and completion of RT/TMZ, prior to initiation of maintenance TMZ, in 46 patients. PFS was 18 months in experimental patients compared to 7.3 and 6.2 months in the two placebo groups [28].

Additionally, BioNTech and Immatics tested personalized TSA vaccines on 15 HLA-A*02:01– or HLA-A*24:02- restricted ndGBM patients with life expectancy greater than 6 months and containing up to 84 non-synonymous mutations and poly-ICLC and GM-CSF as adjuvants. Vaccination with this product induced sustained responses of central memory CD8 T cells and type 1 T helper CD4 T cell responses in 80% of treated patients. The mOS was 29 months with PFS of 14.2 months; one patient had OS > 38.9 months [38]. A similar study and results were published in an adjacent article [39]. In both studies, immune responses were best in patients who received no or minimal corticosteroids. In conclusion, a variety of approaches have demonstrated safety and evidence for some clinical activity in a minority of patients, although which patients may respond could not be predicted.

## IGV-001 immunotherapy treatment: historical background of IGV-001 a biologic-device combination product

IGV-001 is an autologous cancer cell-based immunotherapeutic approach designed to deliver an antigenic payload in the context of immunostimulatory molecules to patients with GBM. IGV-001 consists of autologous GBM cells that are incubated with an antisense oligodeoxynucleotide (IMV-001) targeting insulin-like growth factor 1 receptor (IGF-1R), placed in proprietary biodiffusion chambers (BDCs) with an 0.1 μm pore size and charged with additional IMV-001, then sealed and irradiated. The BDCs are then implanted in patients’ abdominal wall between the rectus sheath and muscle inferolateral to the umbilicus (the lymphatic watershed below possible immunosuppressive GBM-tolerized draining lymph nodes) for approximately 48 h [40]. As opposed to other autologous cancer vaccine modalities, which require multiple dosages over weeks and months [14, 41], IGV-001 consists of only one treatment given within 48 h of craniotomy within a standard of care hospitalization.

Regarding the IGF-1R antisense component IMV-001, previous preclinical research revealed that targeting IGF-1R, a surface receptor activated by its ligand IGF-1, initiates a cascade of downstream pathways that ultimately leads to anti-apoptotic signaling and maintenance of cell viability. Renato Baserga, among others, advanced the theory that upregulation and expression of IGF-1R in cancer is responsible for maintenance of the malignant phenotype and downregulating IGF-1R renders the cell susceptible to apoptosis [42–44]. Data from the Baserga laboratory supported the use of an 18-mer antisense oligodeoxynucleotide to IGF-1R as a therapeutic agent in a rodent model. In studies with ovarian or lung cancer cell lines, this drug class outperformed monoclonal antibodies or small molecule inhibitors when targeting IGF-1R [45].

In the C6 murine glioma model, C6 glioma cells were preincubated with *IGF-1R* antisense oligodendronucleotide, encapsulated in millipore diffusion chambers with a 0.1 μm Durapore® exclusion limit, and implanted in the flank of rats [44]. We adopted this approach in a phase 1a human trial under IND6776, in which viable autologous recurrent GBM cells gathered during tumor re-resection were encapsulated within millipore chambers of the same specification and implanted in the patient’s abdomen on the first postoperative day. The trial confirmed the safety of this paradigm with unexpected clinical responses [46].

## Phase 1b trial evaluating IGV-001 in the treatment of ndGBM

With the later IND14379, the original phase 1a study was replicated and a phase 1b study was completed for ndGBM patients, which confirmed safety and demonstrated both unanticipated treatment response and efficacy endpoints. IGV-001 may be advantageous in that it includes an unselected population of cancer cells, thereby including a broad antigenic signature of a patient’s tumor. In this trial, a typical phase 1 3 + 3 dose-escalation design [47] was not optimal, as toxicity was not likely to be an issue in the same fashion as a typical phase 1 study. Instead, dose escalation was based on four randomized cohorts assigned to receive different numbers of BDCs (i.e., ten or twenty); the combination product was implanted in the abdominal wall for either 24 or 48 h. Notably, the BDCs were dosed only once. Implantation occurred within 24 h of surgery and was followed in 6 weeks by RT/TMZ. Randomization was terminated early due to significant clinical and radiographic response in the highest dose exposure cohort; after patient 23, only the highest dose was administered. Thirty-three total patients were treated, with PFS of 9.8 months as compared to 6.5 months in historical controls receiving SOC. In the sample, 16 patients were MGMT-methylated (48.5%), as compared to 17 patients with unmethylated MGMT status (51.1%). Patients with the highest IGV-001 exposure and Stupp eligibility (i.e. patients < 70 years of age with unilateral disease) had mPFS of 17.1 months and an mOS of 38.2 months [40]. Of note, only one patient in our cohort had IDH-1 mutation. The results of the trial are summarized in Table 1 with comparison to the Stupp SOC findings.

**Table 1:** Summary of outcomes in the phase 1b trial compared to Stupp SOC. Stupp-eligible criteria exclude patients over 70 years old and those with extensive intracranial disease, including bi-hemispheric disease (butterfly glioma) or multi-centric disease. Adapted from Andrews, D.W., et al. [40]

Patients with newly-diagnosed glioblastoma				
IGV-001 102 phase 1b study				
Groups	Total ITT	Highest dose cohort ITT	Stupp-eligible highest dose cohort	Standard of care [65, 66, 67]
N	33	15	10	1,059
MGMT-methylated	16 (48.5%)	7 (41.2%)	5 (50%)	251 (35.8%)*
mOS	17.3 mo	22.3 mo	38.2 mo*	16.2 mo
OS24	39%	50%	60%	30%
PFS6	86%	85%	90%	56%
mPFS	9.8 mo††	17.3 mo†	17.1 mo**	6.5 mo

SOC values were derived by digitizing published Kaplan-Meier curves from published trials from the technique by Guyot et al. [64]

Note that these controls were not matched for patient-specific data.^††^* p* < 0.0004; ^†^*p* < 0.002; ∗∗ *p*< 0.003: ∗ *p* = 0.044; highest dose cohort ITT approached significance at *p*= 0.08

## Follow-on phase 2b study: randomized placebo-controlled double-blinded evaluation of IGV-001

The safety profile in all four dose cohorts in phase 1b was favorable and comparable. In contrast, the radiographic and clinical outcomes were better in the highest dose cohort (20 chambers for 48 h), and 90% of patients were progression-free at 6 months. This dose was therefore carried forward as the treatment dose for the phase 2b trial design with no intent of additional higher dose cohort arms. Also of interest, the coefficient of determination in the uncensored paired events in the ITT yielded a regression curve of median PFS and OS with *p* = 0.91 and *p <* 0.001. Given the substantial impact of PFS on OS, we established PFS at a hazard ratio (HR) of 0.5 as the primary endpoint, with OS at a HR of 0.5 as the key secondary endpoint.

**Fig. 1:**
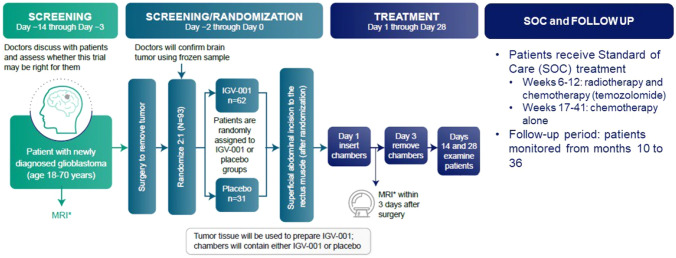
Phase 2B Clinical Trial Design: Patients ages 18–70 with ndGBM are screened and randomized to IGV-001 or placebo prior to surgery. During surgery, glioblastoma is confirmed on frozen section. Superficial abdominal incision is made at the time of surgery to allow for later implantation of biodiffusion chambers. Autologous tumor sample is then used to prepare IGV-001, and either IGV-001 or placebo chambers are implanted postoperative day 1. Chambers are removed on day 3, and patients then proceed with SOC treatment and are monitored from months 10 to 36

In addition to determination of the treatment dose, the phase 1b trial led to production of a commercially-scalable and optimally-designed biologic drug device investigational product that met cGMP standards. The phase 2b trial opened for accrual in March 2023 for patients with ndGBM (NCT04485949, Fig. 1). As an antigen-inclusive and antigen-agnostic platform with the latter two rigorous design clinical trial elements, IGV-001 provides a novel immunotherapy platform for historically intransigent solid tumors in the ongoing phase 2b ImmuneSense trial in ndGBM.

## IGV-001 rationale and mechanism of action

**Fig. 2:**
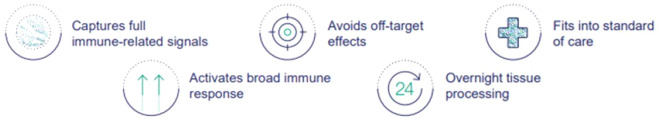
Advantages of The Goldspire^™^ Platform: unique advantages of the Goldspire^™^ platform highlight the benefits of autologous tumor-derived therapy for glioblastoma

IGV-001 is the first product of the Goldspire^™^ platform, which delivers a tumor-derived antigenic payload and immunostimulatory signals that together induce innate and adaptive immunity against residual malignant cells (Fig. 2). Tumors typically contain ten to five hundred protein-changing mutations, 98% of which are unique to each individual solid tumor. Castle et al. mined data from The Cancer Genome Atlas program to assess expressed tumor mutational burden grouped by cancer indication tumor type [48]. In all cases, this represents non-synonymous mutations that change protein structure and are thus immunogenic. This includes peptides bearing the mutation, as well as downstream post-translational modifications of peptides, such as methylation or phosphorylation, adding to the immunogenicity of the antigen payload [49].

Preclinical studies conducted to dissect the mechanism of action (MoA) of IGV-001 corroborated anti-tumor activity in the murine variant of this product, *m*IGV-001, in the GL261-luciferase (GL261-luc) GBM mouse model and detected *m*IGV-001-induced immune responses in the BDC-draining lymph nodes [50]. The GL261 model is one of the major and most utilized in vivo, orthotopic, syngeneic glioblastoma models. Advantages and limitations of this model and others have been described in detail elsewhere [51]. We utilized this model, despite its constraints, because GL261 cells can be implanted into immunocompetent C57/BL6 mice without rejection due to their C57/BL6 background.

Furthermore, these studies suggested that the combination of IMV-001, irradiation [52, 53] of the biologic product in BDCs, tumor dissociation into single cells, lack of supportive extracellular matrix scaffolding [54], and diminished nutrient availability within the BDCs contribute to cell death of autologous tumor cells inside the BDC. While cell death occurs physiologically mainly in an immunosilent manner [54], it can elicit innate and adaptive immune responses [55]. The latter type of regulated cell death is also referred to as “immunogenic cell death” (ICD). ICD can be triggered by various stimuli [56]. As described above, the Goldspire^™^ approach to inducing ICD relies on multifactorial stimuli to generate a tumor antigen payload through ICD of autologous cancer cells to elicit a potent innate and adaptive anti-tumor immune response [50].

**Fig. 3:**
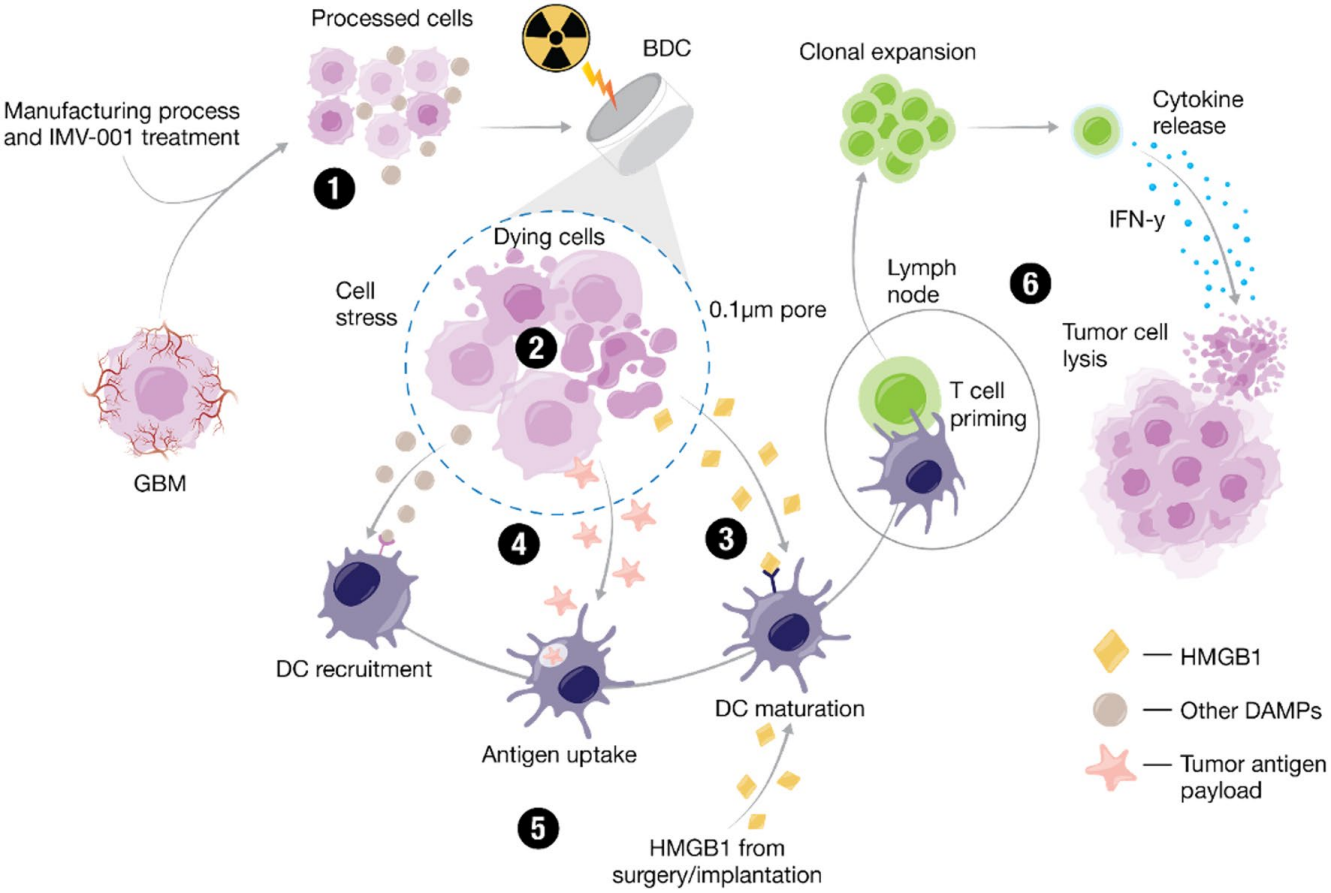
The Goldspire^™^ Platform Proposed Mechanism of Action: (1) After manufacturing process, combination drug product (IMV-001-treated autologous tumor cells + IMV-001) is placed in biodiffusion chambers (BDCs), which are then irradiated and sent to the clinical site for implantation into the abdomen of the patient; (2) due to the irradiation, isolated IMV-001 treatment, low-nutrient environment, and inability to adhere inside the BDC, tumor cells are exposed to cellular stresses that ultimately result in cell death; (3) high mobility group box 1 (HMGB1), and damage-associated molecular patterns (DAMPs) produced during immunogenic cell death (ICD), are released from stressed/dying cells inside the BDCs and from the surrounding damaged tissue at the implantation site; (4) also released from the BDCs is a tumor antigen payload (< 0.1 μm in size); (5) dendritic cells (DCs) are recruited by DAMPs adjuvanticity and mature upon tumor antigen uptake; (6) DC-primed T cells undergo clonal expansion and tumor-antigen specific T cells kill tumor cells. (This figure was created with BioRender.com and then further modified.)

Imvax nonclinical studies [50] using the murine GL261 GBM model and human GBM cell lines in the IGV-001 product have confirmed the release of danger/damage-associated molecular pattern (DAMP) immune stimulators, including adenosine triphosphate (ATP) and high mobility group box 1 (HMGB1) [50, 55, 57, 58], as well as cellular debris/antigenic payload (< 0.1 μm in size) from dead and dying cells within the BDCs (Fig. 3). ATP elicits a “find-me” signal that attracts and activates DCs, whereas HMGB1 promotes DC antigen presentation [59]. Phenotypic evaluation of immune cells in the GBM model showed an increased percentage of DCs as well as effector and effector memory T cells in the draining lymph nodes proximal to BDCs [50].

Furthermore, T cells from mice receiving the murine version of IGV-001 produced IFNg in response to known tumor antigens from murine GL261 GBM cells [50]. Similar evidence of immunologic activity was seen in other mouse cancer models, including ovarian and urothelial cancers and hepatocellular carcinoma [50, 60, 61]. Together, these data strongly suggest that the use of this biologic-drug device product is a suitable approach to generate, contain, and release subcellular antigenic and immunogenic cargo to stimulate the immune system. DC primed with tumor antigens and aided by DAMPs provide co-stimulatory signals that are critical for the generation of cytotoxic anti-tumor-specific T cells [50, 56, 62]. In summary, ICD-associated pathways are believed to be responsible for the immune responses elicited by IGV-001 immunotherapy.

## Beyond IGV-001

Studies conducted in syngeneic murine models support the use of the Goldspire^™^ immunotherapy platform to treat a number of solid cancers beyond GBM. In the ID-8 murine ovarian carcinoma model (intraperitoneal, “metastatic-like”) [60, 61], the Hepa1-6 murine hepatocellular carcinoma model (orthotopic) [50, 62], and the MBT-2 murine bladder cancer model (orthotopic) [61], mice receiving a BDC prepared with the respective tumor cell line experienced significant prolongation of their mOS compared to mice implanted with saline-containing BDCs. These studies also demonstrated that the efficacy of this biologic-device combination product is associated with a systemic and durable immunological response, resulting in generation of Th1 antitumor cytotoxic T cells (unpublished data). Additional testing conducted in subcutaneous murine models of renal cell carcinoma (RENCA), and colorectal cancer (CT26) also showed a beneficial decreased and/or delayed tumor burden in mice treated with this immunotherapy approach compared to control animals (unpublished data).

## Conclusions

Immunotherapy for treatment of solid tumors is a promising concept, particularly in the treatment of GBM, a disease with a poor prognosis despite SOC therapy. Therapeutic cancer vaccines aim to circumvent the substantial challenges to immunotherapy when treating GBM, including the heterogeneity of tumors within and between patients, the immune-privileged nature of the central nervous system, and the immunosuppressive environment created by the tumor, as well as the radiation, chemotherapy, and corticosteroids that comprise standard treatment. Beyond the 2005-published Stupp trial [24], phase 2 clinical trials for GBM typically have been single-arm trials that have failed to meet primary clinical endpoints. In a broader and recent analysis of 3038 neuro-oncology clinical trials, most trials were nonblinded (92%), single group (65%), nonrandomized (51%) and almost half were GBM trials [63]. Only 10% involved a biologic and only 2.2% involved a double-blind randomized trial design. Breakdown of these data, including nearly 300 blinded trials and 60 double blind randomized trials, shows that only six were cancer vaccine-like approaches. Further phase 2 and 3 trials with rigorous study design are needed to continue to advance cancer vaccine approaches to GBM.
