# Correction to: Targeted immunotherapy for glioblastoma involving whole tumor-derived autologous cells in the upfront setting after craniotomy

**DOI:** 10.1007/s11060-024-04635-0

**Published:** 2024-03-22

**Authors:** Carrie E. Andrews, Jenny Zilberberg, Raul Perez-Olle, Mark A. Exley, David W. Andrews

**Affiliations:** 1 Department of Neurological Surgery, Thomas Jefferson University, 19107 Philadelphia, PA USA; 2 Imvax, Inc, 19602 Philadelphia, PA USA

**Correction to: Journal of Neuro-Oncology (2023) 165:389–398**

10.1007/s11060-023-04491-4

The article ‘Targeted immunotherapy for glioblastoma involving whole tumor-derived autologous cells in the upfront setting after craniotomy’, written by Carrie E. Andrews, Jenny Zilberberg, Raul Perez‑Olle, Mark A. Exley and David W. Andrews, was originally published Online First without Open Access. After publication in volume 165, issue 3, pages 389–398, the author decided to opt for Open Choice and to make the article an Open Access publication. Therefore, the copyright of the article has been changed to © The Authors 2023 and the article is forthwith distributed under the terms of the Creative Commons Attribution 4.0 International License, which permits use, sharing, adaptation, distribution and reproduction in any medium or format, as long as you give appropriate credit to the original author(s) and the source, provide a link to the Creative Commons licence, and indicate if changes were made.

The images or other third party material in this article are included in the article’s Creative Commons licence, unless indicated otherwise in a credit line to the material. If material is not included in the article’s Creative Commons licence and your intended use is not permitted by statutory regulation or exceeds the permitted use, you will need to obtain permission directly from the copyright holder.

To view a copy of this licence, visit http://creativecommons.org/licenses/by/4.0/.

